# Dataset on electrical single-family house and heat pump load profiles in Germany

**DOI:** 10.1038/s41597-022-01156-1

**Published:** 2022-02-15

**Authors:** Marlon Schlemminger, Tobias Ohrdes, Elisabeth Schneider, Michael Knoop

**Affiliations:** 1 Institute for Solar Energy Research Hamelin (ISFH), Am Ohrberg 1, 31860 Emmerthal, Germany; 2 Department Solar Energy, Leibniz University Hannover, Appelstr. 2, 30167 Hannover, Germany

**Keywords:** Energy science and technology, Electrical and electronic engineering

## Abstract

This paper describes a dataset of residential electricity household and heat pump load profiles, measured in 38 single-family houses in Northern Germany. We provide data per household of apparent, active and reactive power (W), voltage (V), current (A) and the power factor (no unit) in 10 seconds to 60 minutes temporal resolution from May 2018 to the end of 2020. We validated the dataset both in itself, comparing different measurements that should produce the same results, and externally to standard load profiles and found no major inconsistencies. We identified an average consumption per single-family house with 2.38 inhabitants of 2829 kWh for the household and an additional 4993 kWh for the heat pump. The dataset can support the understanding of patterns in electrical load curves and can help to estimate the additional load on distribution networks induced by heat pumps.

## Abstract (summary)

**Table:** 

Measurement(s)	household power • heat pump power • voltage • current
Technology Type(s)	direct connected meter
Sample Characteristic - Environment	household
Sample Characteristic - Location	Germany

Machine-accessible metadata file describing the reported data: 10.6084/m9.figshare.17206271

## Background & Summary

Residential buildings are a major contributor to energy consumption. In the European Union, the residential sector accounted for 26.1% of the final energy consumption in 2018, of which 78.4% are used for space and water heating and the remaining 21.6% are used for electric end-uses such as lighting^1^ or appliances. The share of electricity consumption is expected to increase with the rising relevance of heat pumps.

Measurements of electric load profiles can contribute to understanding patterns in the consumption and identifying load shift potentials. Here, we present a dataset, covering measurements, which were carried out within the framework of the research project Wind-Solar-Heat Pump District (WPuQ) from 2018 to 2020 in 38 single-family households in a district in Lower-Saxony, Germany. We provide measurements of the electrical household load for three phases of voltage, current, active, apparent and reactive power in a 10-second resolution. Additionally, separate measurements of electrical loads of the heat pumps installed in each building and of the electrical substation with the aggregated load of 68 households in total are available.

Various public datasets with similar purpose are available. The University of Applied Sciences Berlin published a dataset of 74 households’ electrical load profiles in Germany from 2010 in one-second resolution^2^. Trindade made available measurements across 370 households in Portugal from 2011 to 2014 in 15-minute resolution^3^. Hebrail’s dataset contains measurements from a single household in France from 2006 to 2010 in a one-minute resolution^4^. Makonin *et al*. published measurements from a single household in Canada from 2012 to 2014 in a one-minute resolution^5^. Smart-meter data of over 5000 households in the UK measured between 2011 and 2014 in a 30-minute resolution is available via the UK Power Networks^6^. Kleiminger provides a dataset measured across six household in Switzerland over 8 months in one-minute resolution^7^. Kelly published a dataset on the appliance level measured across five households in the UK at a temporal resolution of 16 kHz^8^. The dataset of Barker *et al*. contains detailed smart-home measurements of three households for multiple years and load profiles of more than 400 households for a single day^9^.

Despite the large amount of datasets available, there is space for improvement in each of them: Most datasets are measured in a single-digit number of households^4,5,7–9^ and some datasets only provide a temporal resolution of 15 or more minutes^3,6,9^. Additionally, measurements including electrical properties such as voltage, current or reactive power are missing^2–4,6^ and some datasets only make available their data, but are not published in a scientific journal^3,4,6^. None of the datasets provides measurements of electrical household loads and heat pump loads with corresponding weather data.

The WPuQ dataset^10^ is able to fill the gaps left by the aforementioned datasets and provides a unique combination of a large sample size, high number of measured indicators, high temporal resolution and long measurement period.

## Methods

### Buildings

**Fig. 1:**
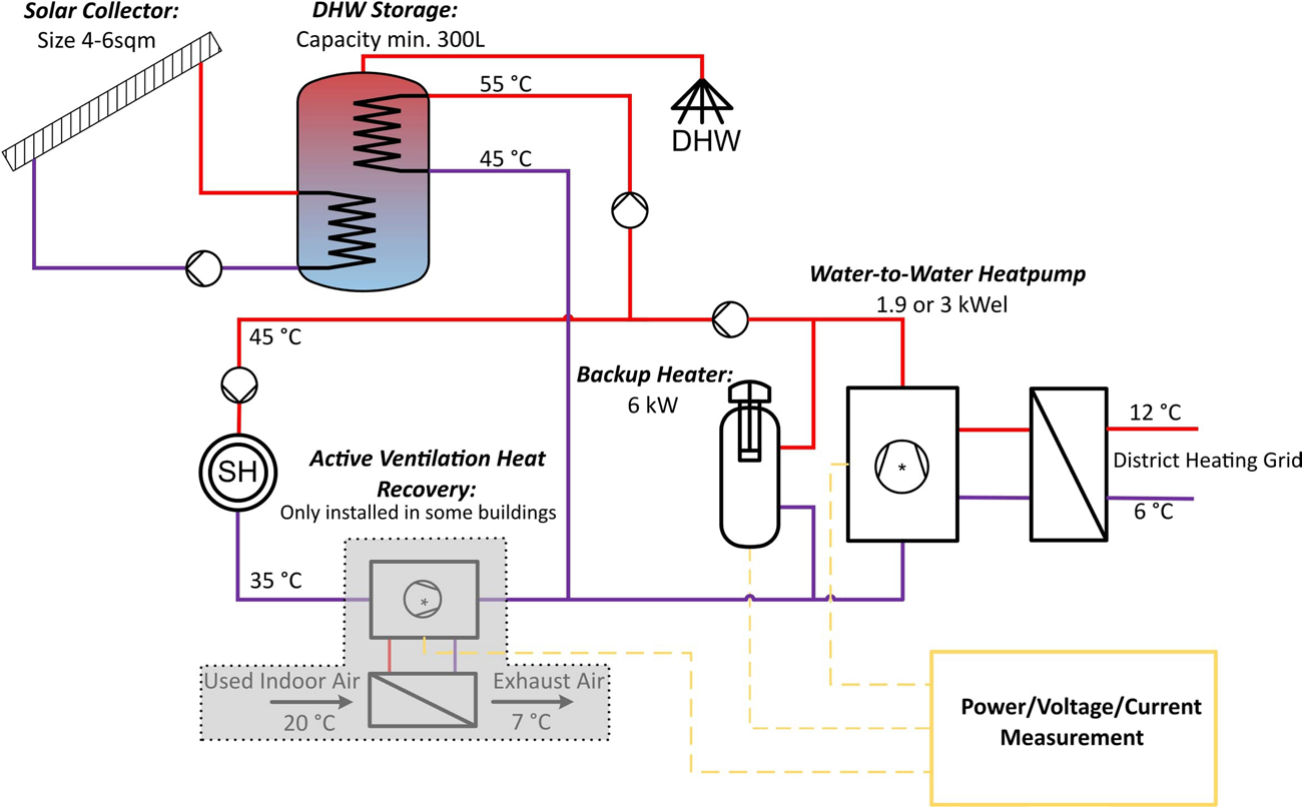
Hydraulic scheme of the heat pump system in the buildings. The ventilation system marked in gray is present in only a few buildings. The electricity meter records the electricity for the heat pump, the heating element, the heat pump control and, if applicable, the ventilation system.

The measurements are part of the WPuQ project and take place in an enclosed district near Hamelin in Lower Saxony, Germany. The district consists of 68 single-family houses built in the late 90 s and early 2000s and all houses comply with the low-energy standard with a specific heat demand of about 45–50 kWh/(m^2^a)^11^. All buildings are equipped with water-water-heat pumps connected to a cold local heating network and solar thermal systems for domestic hot water (DHW), as shown in Fig. 1. The heat pumps have 7.4–11.3 kW thermal power at an operating point of 10 °C inlet temperature on the primary side and 35 °C outlet temperature on the secondary side and are equipped with a 6 kW heating rod as backup heater. The nominal electrical capacity of the heat pump compressor is either 1.9 or 3 kW_el_. The cold local heating network provides the heat pumps with 10 to 12 °C water. Underfloor heating provides the space heat (SH). The solar thermal systems with 4–6 sqm collectors provide DHW mainly in summertime. If there is not enough solar energy available, the heat pump takes over the DHW production. Few buildings are additionally equipped with a ventilation system with heat recovery and PV systems. All building parameters are summarized in Table 1.

**Table 1:** Characteristics of the measured households.

Building number	Building area [m²]	Number of inhabitants	PV system size [kWp]	Ventilation system
3	140	2	—	—
4	160	2	—	—
5	160	3	—	Yes
6	140	1	—	—
7	150	2	—	—
8	160	2	—	—
9	195	4	—	Yes
10	135	3	—	—
11	230	4	—	—
12	112	2	—	—
13	130	2	2.5	—
14	150	2	—	—
15	120	1	4.5 (+4.3 kWh battery storage)	—
16	136	4	—	Yes
17	200	2	—	—
18	87	2	—	—
19	203	2	—	—
20	220	4	—	—
21	110	1	—	—
22	117	4	—	—
23	113	2	—	—
24	120	4	—	—
25	100	3	—	—
26	120	1	0.75 (estimated)	—
27	110	1	—	—
28	145	3	—	—
29	104	2	—	—
30	—	3	—	—
31	135	2	—	—
32	160	4	—	—
33	111	2	4	—
34	110	1	—	—
35	100	3	—	—
36	108	2	—	—
37	199	2	—	—
38	190	2	—	—
39	135	2	—	—
40	120	2	—	—

### PV system

In order to be able to determine not only the energy consumption in the district but also the simultaneous local electricity generation from renewable energy sources, we additionally measure three PV systems with different orientations and tilt angles near the district. The PV systems consist of *Solarfabrik Vision 60 poly* modules with 250 W_peak_ nominal power and 15.1% module efficiency. The configurations for each PV system are shown in Table 2. The south-oriented system is representative in terms of orientation and slope for PV systems on the buildings.

**Table 2:** Configurations of the PV systems close to the district.

Name	Orientation (0° = south)	Tilt angle	Nominal power in kW_peak_	Number of Modules	Inverter
East	−83°	10°	19.5	78	Fronius Symo 20.0-3-M
West	97°	10°	19.5	78	Fronius Symo 20.0-3-M
South	−5°	30°	14.5	58	Fronius Symo 15.0-3-M

We use the south-oriented system to estimate electricity generation of the buildings in the district with PV panels installed.

### Measurement of household and heat pump load

**Fig. 2:**
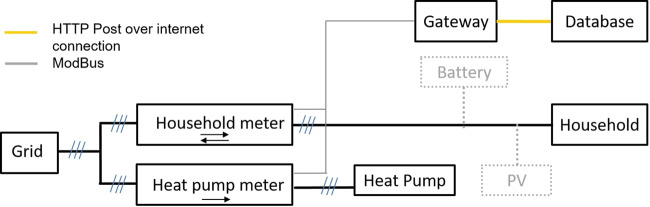
Structure of the measuring technology installed in each household. There are two separate meters measuring the household and heat pump load. PV systems may directly feed into the household circuit and are therefore invisible to the meter.

We perform separate measurements in 38 households and an aggregated measurement at the electrical substation supplying all 68 households. The installation of measuring technology started in 2017 and data is available for most households starting from May 2018. Figure 2 shows the structure of the measuring technology installed in each building. We installed separate power meters for the household and the heat pump load. Electricity for pumps in the heating circuit and the solar thermal system are recorded via the heat pump meter. In the case of a ventilation system, this is also recorded by the heat pump meter.

If the building is equipped with a PV system with self-consumption (see Table 1), the PV system is connected to the household circuit. Therefore, the measurement of the household load is superimposed by the feed-in of the PV system.

We measure the household and heat pump electricity consumption on three phases with direct connected meters (*ABB, type B23 312-100 Silver*, Accuracy Class 1 for active power and Accuracy Class 2 for reactive power^12^). The measurements in the substation are performed on three phases by means of transformer connected meters (*ABB, type B24 212-100 Bronze*, Accuracy Class 1 for active power and Accuracy Class 2 for reactive power^13^) with external current transformers (*KBU, type 812 1200/5* *A*, Accuracy Class 0,5). A *Symcon Symbox Neo* with *RS485 ModBus RTU interface*^14^ serves as data logger and gateway. The *Symbox* reads out the instantaneous values as well as the meter readings of the electricity meters in intervals of 10 seconds via *ModBus RTU* and stores them provided with a time stamp from the build-in real time clock in a buffer. Every 1–5 minutes, the *Symbox* transmits the buffered data via the buildings internet connection (DSL/cable) by HTTP POST requests to the database server.

### Measurement of photovoltaic power generation

In the PV systems, the inverters measure the feed-in power at the AC-side with integrated meters and provide the values via *ModBus TCP SunSpec* interface. As in the buildings, we use a *Symbox* to read out the data via *ModBus TCP* connection at intervals of 10 seconds and forward them to the database server.

### Measurements in the district heating grid

The flow and return temperatures of the local heating network are measured by means of contact temperature sensors (*1-wire DS18S20*^15^, accuracy ±0.5 °C from −10 °C to +85 °C) in the heating centre. The temperature sensors are connected to an *ESERA 1-Wire Gateway 11 Modbus TCP*^16^. As previously mentioned, we use a *Symbox* to read out the sensor data over *ModBus TCP* at intervals of 10 seconds and forward them to the database server.

### Weather data

We obtain weather data such as outdoor air temperature, wind speed, relative humidity and solar global radiation from the weather service wetter-online^17^ for the location Hamelin. We request the current weather data in intervals of 5 min from the weather service via a HTTP REST API and store it on the database server. The year 2018 has irregular intervals of 1 min to 1 hour.

### Data processing

We use the *emoncms*^18^ system as the central database server. The system receives the data from the gateways, performs the authentication and stores the measurement data in a MySQL database. The raw data stored in this way is then validated (section Technical Validation), aggregated and stored in HDF5 format (section Data Records). The data processing code is available at https://github.com/ISFH/WPuQ.

## Data Records

All data are available on the Zenodo platform^10^. The total data consists of seven hierarchical HDF5 files per year, of which five files contain our measurements and two additional files contain weather data and temperatures of the district heating network nearby. Each of the five files with our measurement data provides similar information in different aggregations. The structure of each file is shown in Table 3. We aggregate data to four different temporal resolutions (10 s, 1 min, 15 min and 60 min) for convenience reasons and provide a file where we spatially aggregate the profiles of all households into single profiles. The original sample interval is 10 s. Naturally, an HDF5 file orders datasets into groups, where each group is analogous to a file system directory. In our case, each group consists of up to three nodes (top-, middle- and lower-level nodes), splitting the data into categories.

**Table 3:** Structure of the datafiles, exemplary for the resolution of 10 s and the spatially aggregated file.

Filename	Top-level nodes	Middle-level nodes	Low-level nodes	Datasets
**data_10 s**	MISC	ES1	TRANSFORMER	S, P, Q, PF, U, I
		PV1	EAST	S, P, Q, I, U
			SOUTH	
			WEST	
	NO_PV	SFH3	HOUSEHOLD	S, P, Q, PF, U, I
			HEATPUMP	S, P, Q
		…	HOUSEHOLD	S, P, Q, PF, U, I
			HEATPUMP	S, P, Q
		SFH40	HOUSEHOLD	S, P, Q, PF, U, I
			HEATPUMP	S, P, Q
	WITH_PV	…	…	…
**data_spatial**	SUBSTATION	10s	—	P, Q
		…	—	P, Q
		60 min	—	P, Q
	NO_PV	10 s	HOUSEHOLD	P, Q
			HEATPUMP	P, Q
		…	HEATPUMP	P, Q
		60 min	HOUSEHOLD	P, Q
			HEATPUMP	P, Q
	WITH_PV	…	…	…
**weather**	WEATHER_SERVICE	IN	..TEMPERATURE..	TEMPERATURE
			..WIND_SPEED..	WIND_SPEED
			…	…
**dh_grid**	DH_GRID	IN	..FLOW	TEMPERATURE
			..RETURN	TEMPERATURE

The top-level nodes primarily split the data into households with and without PV own consumption. As explained in the section Methods, PV own consumption tampers with the measurements, because it is invisible to the meter. For these buildings, the measured values are the sum of the actual household consumption and the production of the PV system (with a negative sign) and can therefore become negative during times when the house is a net producer. We append a “WITH_PV” to the active power measurements that include PV feed-in. Additionally, we estimate the household’s PV production by its installed capacity and the capacity factors measured at another PV system close-by. We use this estimate to provide a corrected active power curve that excludes the PV production. The third category of top-level nodes is MISC/SUBSTATION, where we provide the measurements of the PV system close-by and the measurements of the electrical substation.

The middle-level nodes of the files in different temporal resolutions provide the data for each single-family house (SFH) followed by a number corresponding to the building number shown in Table 1. Additionally, we provide nodes for the electrical substation (ES1) and the PV system (PV1). The middle-level nodes of the spatial file contain the four temporal resolutions.

The low-level nodes contain the differentiation between household and heat pump load. For the PV system, different feed-in for east, west and south orientation are included.

Both the weather data and the district heating grid file only contain a single top- und middle-level node. The low-level nodes store the different weather parameters such as ambient temperature or wind speed and the flow and return temperature of the district heating grid.

Each group, which is a unique combination of a top-, middle- and low-level node, contains a two-dimensional array of data elements (columns and rows). The first column is always the date and time of the measurement in seconds since 1970 (UNIX timestamp) while the following columns show measurement results. We always build mean values when we resample between temporal resolutions. This means that for example active power measurements in a temporal resolution of 10 s describe the average power during that time interval. A detailed explanation of each column description is given in Table 4.

**Table 4:** Description of dataset columns.

Column header	Appended Information	Unit	Description
index	—	Seconds since 01.01.1970 00:00:00 UTC	Date and time of the measurement.
S	_1/_2/_3/_TOT	VA	Apparent power. An appended number signals measurements on a single phase (1, 2 or 3) and appended “TOT” signals the sum over all phases. An appended “_TOT_WITH_PV” signals a measurement including PV feed-in.
P	_1/_2/_3/_TOT/_TOT_WITH_PV	W	Active power. An appended number signals measurements on a single phase (1, 2 or 3) and appended “TOT” signals the sum over all phases.
Q	_1/_2/_3/_TOT	VAR	Reactive power. An appended number signals measurements on a single phase (1, 2 or 3) and appended “TOT” signals the sum over all phases.
U	_1/_2/_3	V	Voltage. An appended number signals measurements on a single phase (1, 2 or 3).
I	_1/_2/_3	A	Current. An appended number signals measurements on a single phase (1, 2 or 3).
PF	_1/_2/_3	—	Power factor. An appended number signals measurements on a single phase (1, 2 or 3).

An appended number signals a phase while an appended “TOT” signals the sum over all three phases. An appended “TOT_WITHOUT_PV” signals that we estimated a theoretical load curve without own consumption of PV systems.

## Technical Validation

In this section, we visualize the dataset and validate it by showing the consistency in itself and comparing it to other statistics. We will focus on the year 2019 when presenting annual statistics, but we ensured that the years 2018 and 2020 show similar behaviour.

### Data gaps

**Fig. 3:**
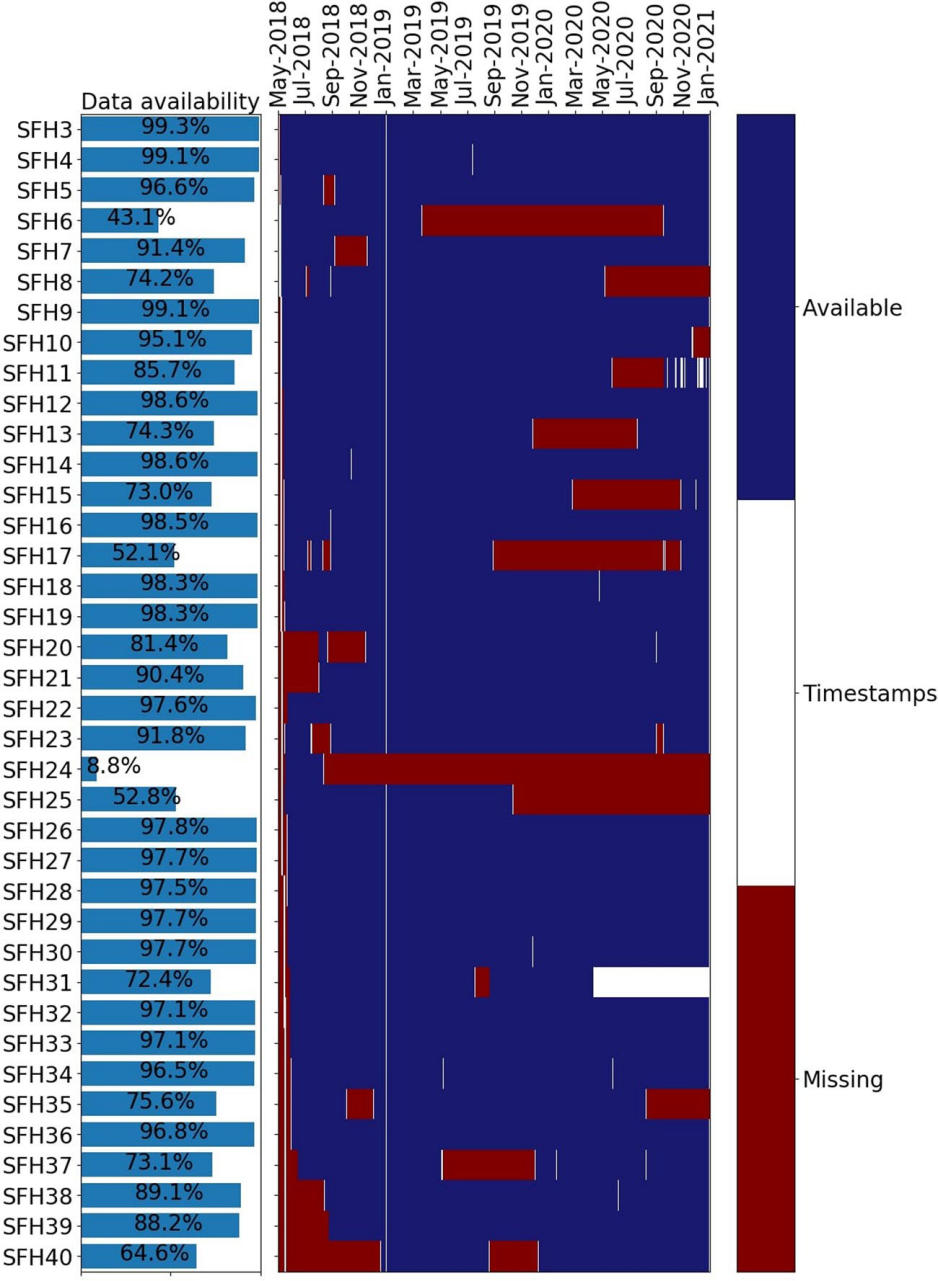
Data availability for the household measurements of all single-family houses. Blue entries mark available data, red entries mark unavailable data and white entries mark interpolated data of up to one day. White zones appearing larger than one day are a visual phenomenon due to the limited amount of pixels available for the plot and in reality have data available in-between.

Fig. 3 plots the missing data for each household. The left side shows the average percentage of data availability for each household and the right side shows the timeline when data is available or missing. We achieve a data availability larger than 90% for 23 out of the 38 households and only two households have an availability of less than 50%. The largest data gaps arise at SFH24, where data is missing from September 2018 onwards.

We correct small data gaps of up to one day by linear interpolation between the nearest known values and mark these interpolations as *Timestamps* in white. We do not interpolate data gaps larger than one day and mark these missing values as *Missing* in red. The according timestamps marked as red are *not-a-number (NaN)* in the published files. The reasons for missing data are mostly technical failures of the data logger or a long disconnection from the internet leading to a buffer overflow and data loss. In two cases, the measurement equipment was permanently decommissioned by the building owners due to the sale of the building or conversion work.

In April 2020, the household *SFH31* started to report values sporadically in intervals of multiple minutes. In Fig. 3, it looks like there are longer periods of multiple weeks where we interpolate values for this household. However, there are data points available in-between, but they are missing in the plot due to the limited amount of pixels mapped to each data point.

### Internal consistency of power and energy

While only power measurements are included in the published WPuQ datasets, the controllers additionally record cumulative apparent, active and reactive energy. We calculate the average daily power imports between the first timestep t_1_ and the last timestep t_2_ from the energy measurement streams by

1 $${E}_{Avg}(d)=\frac{{E}_{{t}_{2}}-{E}_{{t}_{1}}}{{t}_{2}-{t}_{1}}$$

and the average daily power imports from the power measurement streams by

2 $${P}_{Avg}\left(d\right)=\frac{1}{{t}_{2}-{t}_{1}}{\int }_{{t}_{1}}^{{t}_{2}}P\left(t\right)\,dt.$$

Imports means that we only consider electricity flows into the households and ignore electricity going out from PV production. We compare them to each other and provide the 5th- and 95th-percentiles of the relation between power and energy in Table 5. A relation of one indicates the ideal state where power and energy measurements are identical.

**Table 5:** 5th- and 95th-percentiles of the relation between daily power and energy measurements in the year 2019, split by power (apparent, active and reactive) and feed (household, heat pump and transformer).

	Percentile	S	P	Q
**HOUSEHOLD**	**0.05**	0.996	0.992	0.790
**HOUSEHOLD**	**0.95**	1.017	1.010	1.188
**HEATPUMP**	**0.05**	0.996	0.992	0.993
**HEATPUMP**	**0.95**	1.007	1.010	1.008
**TRANSFORMER**	**0.05**	0.999	0.998	0.998
**TRANSFORMER**	**0.95**	1.011	1.000	1.017

The ideal state is a relation close to one.

The transformer performs best with all percentiles close to one and only a small number of outliers. The percentiles of apparent and active power measurements for the household and heat pump are within ±1.7%, which we consider valid. Reactive power measurements show the largest deviations where the 5th-percentile of the household is 0.79 and the 25th percentile is 0.995. The maximum daily consumption is at 108.5 kWh for the household and 132.4 kWh for the heat pump. For both combined, 99% of the consumption is smaller than 75.5 kWh/d and the median is at 8.4 kWh/d.

### Annual consumption

**Fig. 4:**
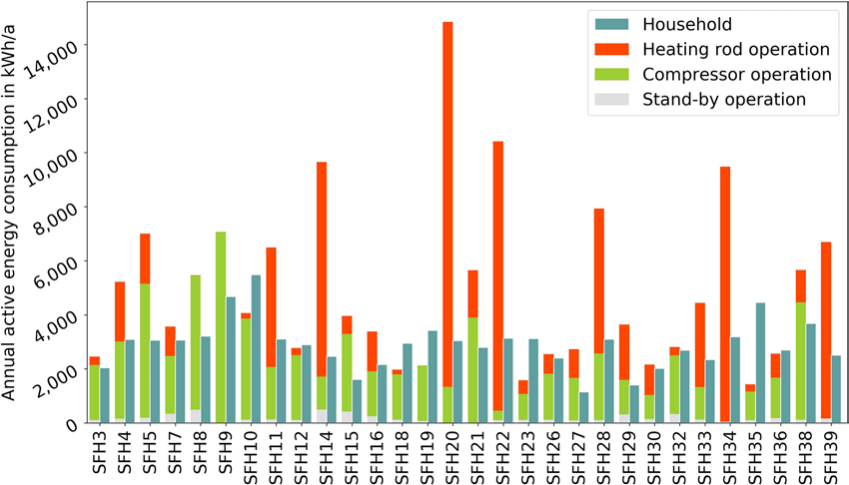
Annual electricity consumption in 2019, separated by the heat pump (left bar) and household (right bar). The households 6, 13, 17, 24, 25, 31, 37 and 40 are missing data for at least one full month. The average consumption of the households without missing data is 2829 kWh/a for the household electricity and 4993 kWh/a for the heat pump. Heat pump operation modes are estimated.

Furthermore, we calculate annual electricity consumption and show it in Fig. 4. We exclude the houses 6, 13, 17, 24, 25, 31, 37 and 40 from the following calculations, because they are missing values for at least one full month and could distort the statistic. According to the German Federal Statistics Office, the average 2-person household in 2018 consumed 3221 kWh and 3+-person households consumed 4978 kWh electricity^19^. 3+-person households have 3.65 inhabitants on average^20^. Our households show an average of 2.38 inhabitants, leading to an expected annual consumption of 3625 kWh by linear interpolation. The average share of electricity for space heating and hot water on this is 17.9%^21^. We exclude this share from our calculation, because we have separate measurements for heat production, leading to an expected annual consumption of 2976 kWh. The remaining 30 households in this work consume an average of 2829 kWh excluding the electricity for the heat pump. The median of the annual household consumption is at 2996 kWh, the minimum at 884 kWh (SFH15) and the maximum at 5489 kWh (SFH10). SFH15 has 4.5 kWp of PV panels and a 4.3 kWh battery storage installed and is therefore expected to have a lower grid supply.

The average heat pump consumption is 4993 kWh with a median at 4012 kWh, the minimum at 1431 kWh (SFH35) and the maximum at 14840 kWh (SFH20). Assuming a heat pump seasonal performance factor (SPF) of 2–3 results in an average heat demand of 10000–15000 kWh. This is lower than the average heat consumption for single-family houses in Germany of 19881 kWh^22^, but to be expected given the age of the quarter of only 20 to 25 years and the generation of DHW by solar thermal collectors. The SPF is comparably low due to technical failures of the heat pumps that increased the operating hours of the heating rod.

The measured data itself does not provide any direct information if heat pumps are operating in compressor mode or if the heating rod supplies the heat demand. Nevertheless, the compressors of the heat pumps have a nominal electrical capacity of 1.9 or 3 kW while the heating rods have a nominal capacity of 6 kW. Both the heating rod and the compressors are not modulating, but deviations from the nominal capacity are expected at different operating points. Therefore, we assume a threshold of 4 kW active power consumption to differentiate between heating rod and compressor mode: we classify timesteps with more than 4 kW active power as heating rod operation and timesteps with less than 4 kW active power as compressor mode. Additionally, we assume that timesteps with an active power consumption with less than 100 W is stand-by operation. From these assumptions, we calculate an energy share of the compressor mode over the whole measurement period of 53.5%, 42.4% for the heating rod and 4.1% for the stand-by on the total active power consumption. Looking at the single-family houses seperately shows notable differences: the five households with the highest heat pump consumption shown in Fig. 4 (SFH14, SFH20, SFH22, SFH34, SFH40) show a share of the heating rod of 53% to 88%. In contrast, there are 12 houses with a share of the heating rod of less than 15%. We advise users of the dataset to be aware of this information while interpreting the data.

### Daily consumption and temperatures

**Fig. 5:**
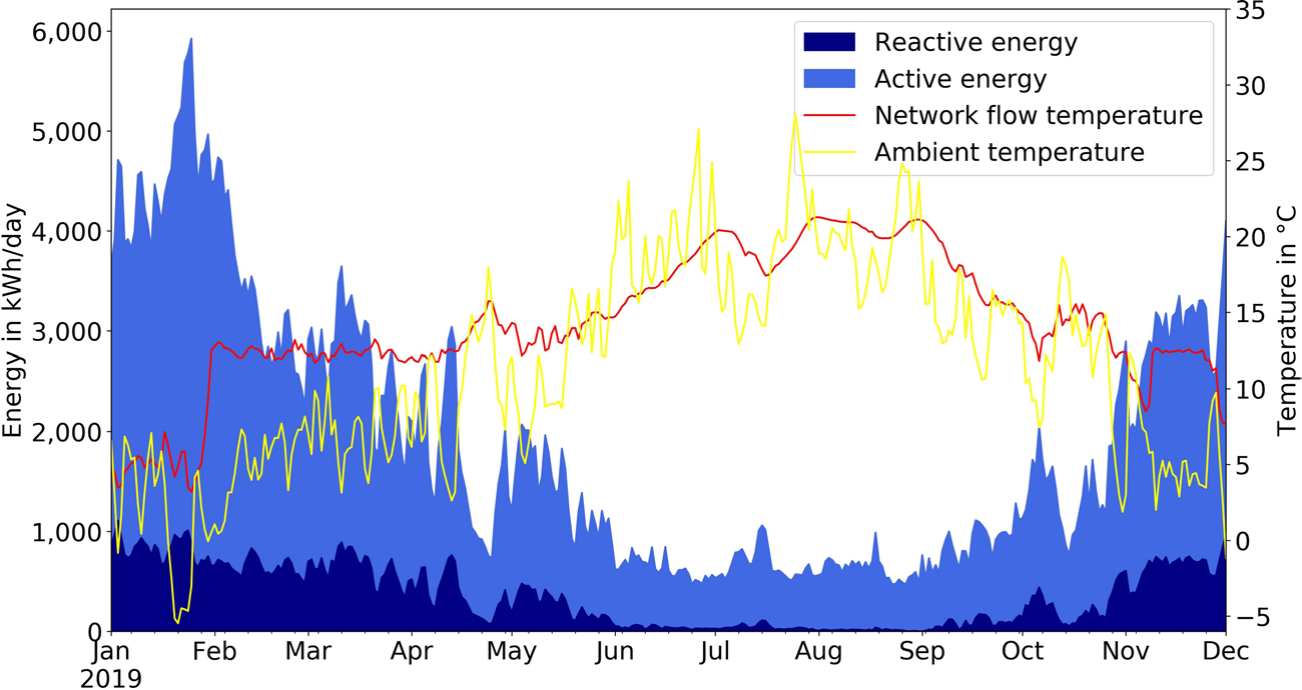
Daily active and reactive energy consumption of the whole district and daily average local heating network flow and ambient temperature. The heat generation of the local heating network failed until the end of January, which decreased its flow temperature to 5 °C.

We plot the daily active and reactive energy consumption P_Avg_ at the electrical substation in combination with the flow temperature of the local heating network and the ambient air temperature in Fig. 5. The electricity consumption of the district fluctuates with a strong negative correlation to the ambient temperature (Pearson correlation coefficient of −0.913) due to the high share of heat pumps on the total consumption. A decrease in temperature such as during late January or early December leads to peaks in the electricity consumption while increasing temperatures such as during late April lead to lower consumption. Interestingly, the reactive energy fluctuates with the active energy while consumption is low, but is capped around 1000 kWh/day. The heat pumps in compressor operation with a power factor smaller than one can only supply 7.4–11.3 kW thermal power. Therefore, the backup heating rods supply high space heating demand, which is an ohmic resistance.

The heat generation of the local heating network failed between mid-December 2018 and late-January 2019, which lead to a flow temperature of 5 °C compared to the usual 10 °C to 12 °C. This increased the electricity consumption of the heat pumps during this period.

### Internal consistency of current, voltage and power factor

**Fig. 6:**
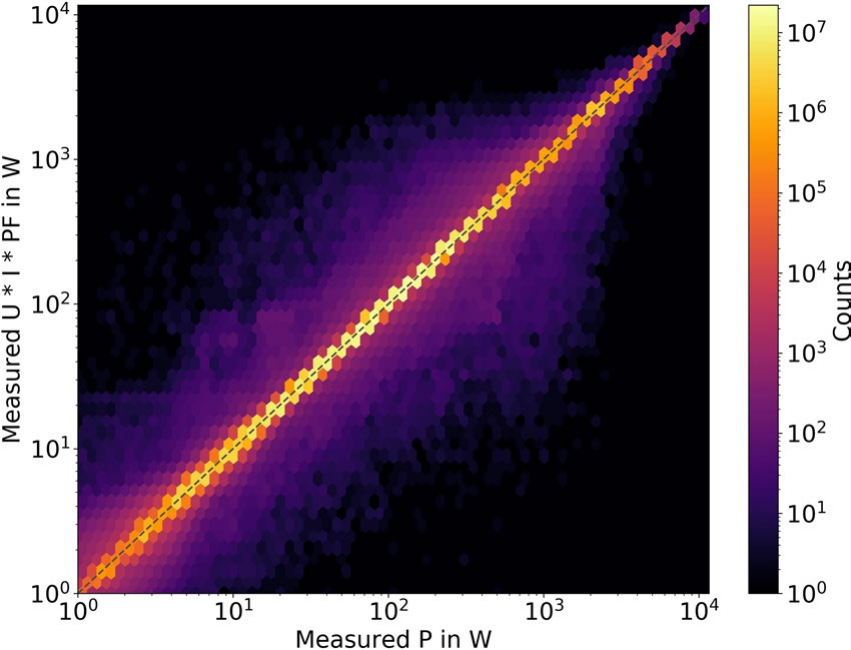
Comparison of measured P and U * I * PF for households in 10 s intervals in the year 2019. We expect both measurement streams to be identical and mark this ideal state by a grey dotted diagonal. We plot both the colouring of the hexagons and the axes on logarithmic scales.

With the validations performed in the paragraphs above, the power measurements are deemed to be valid. We now compare the active power measurements to the datasets of voltage, current and power factor for each phase. Additionally, we do not only consider imports to the households, but look at the absolute value of power regardless of the direction. By

3 $$P=U\times I\times PF$$

we can construct a plot, where we plot the active power on the x-axis, the product of voltage, current and power factor on the y-axis and the ideal state as a diagonal dotted grey line in Fig. 6. We perform this validation on the original temporal resolution of 10 s. The colour of each hexagon correlates to the number of data points in it on a logarithmic scale. The vast majority of value pairs is close to the ideal line, with only some white noise around it. The 5th-percentile of the relation between both measurements is at 0.981 and the 95th-percentile is at 1.023. The maximum load is 11.6 kW, the 99th-percentile is at 3.08 kW and the median is 210 W. Overall, both measurements highly correlate and we did not identify any major inconsistencies in these measurements.

### Compliance with manufacturer data

Additionally, we checked if the measured values are within the range given by the manufacturer of the meters. Measurements outside the range or at its edge would suggest a wrongly designed measurement infrastructure. We found all measurements to be well within the given range though.

### Household load curve

**Fig. 7:**
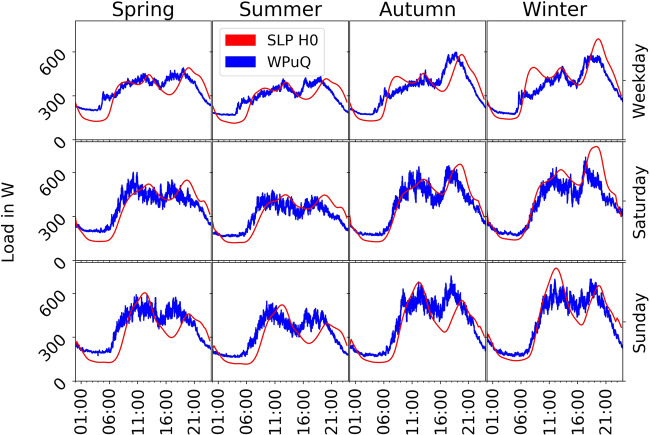
Average household load curve of the WPuQ dataset compared to the German SLP H0, grouped by season, weekday and time. We only plot households without PV production and without missing data, resulting in 27 households. Both curves are scaled to the same annual consumption of 2986 kWh/a for comparison.

In Fig. 7, we plot the resulting active power load curve of the household electricity consumption in comparison to the German standard load profile (SLP) H0 that is commonly used by public services to estimate the load of non-metered customers^23^. We scale the H0 profile to the same annual consumption of 2986 kWh/a as our own measurements. The plot shows the average of all households without PV panels and without missing data, which is 27 households, grouped by season, weekday and time. The general shape of both profiles is similar with a night setback and peaks during the day and in the evening, but major differences exist. It is conspicuous that the shape of our measurements is shifted backwards by approximately 2 hours. The night setback ends around 5 am compared to 7 am and the evening peak is around 5 to 6 pm compared to 8 pm. The load during night is higher (around 170 W compared to 130 W), but lower in the winter evening peak (around 600 W compared to 680 W). Interestingly, the load on Saturdays and Sundays is very similar in our dataset, whereas the SLP handles them differently.

### Heat pump load curve

**Fig. 8:**
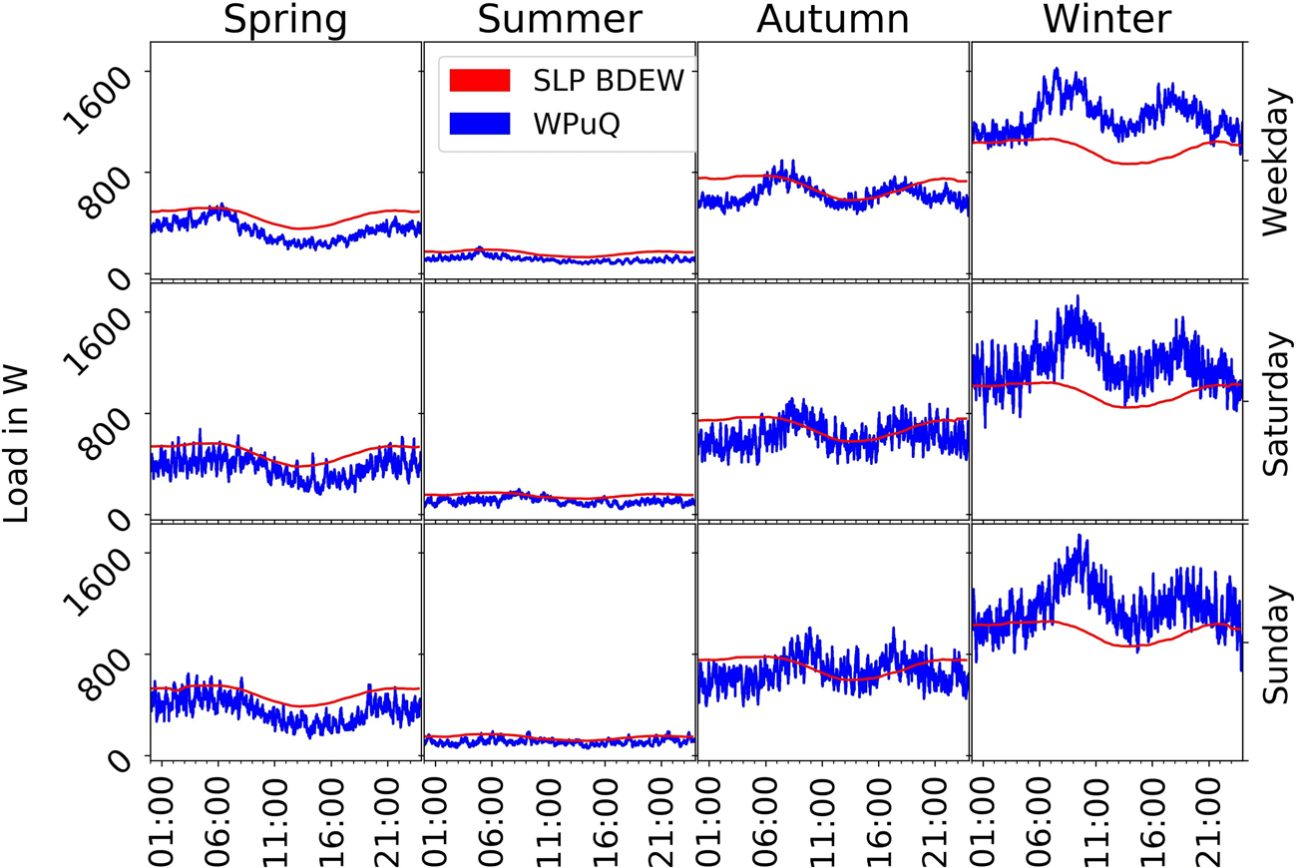
Average heat pump load curve of the WPuQ dataset compared to the SLP of the German Association of Energy and Water Industries (Bundesverband der Energie- und Wasserwirtschaft; BDEW), grouped by season, weekday and time. We only plot households without missing data, resulting in 30 households. Both curves are scaled to the same annual consumption of 4993 kWh/a for comparison.

In Fig. 8, we plot the resulting electric heat pump load curve in comparison to the SLP of the German Association of Energy and Water Industries (BDEW)^24^. We use temperature data of an online provider to calculate the SLP and scale it to the same annual consumption of 4993 kWh/a as our own measurements. It should be noted that the buildings are equipped with a solar thermal system for domestic hot water. The heat pump is therefore only used to heat domestic hot water when solar radiation is insufficient. The space heating is completely generated by the heat pump. Once again, the general shape of both curves is similar. The load in summer is low and almost constant around 180 W. The load increases in both spring and autumn to a level of 500 to 800 W and shows a small trough during the day. The profile during winter deviates the most between both profiles. The SLP assumes a relatively constant load around 1000 W, whereas our measurements show larger fluctuations between 700 and 1800 W and a 23% higher consumption in winter overall. This might be caused partially by the failure of the local heating grid in January 2019.

## Usage Notes

The WPuQ dataset is publicly available via 10.5281/zenodo.5642902 and contains seven HDF5 files per year (21 files in total), of which five files contain our measurements and two additional files contain weather data and temperatures of the district heating network nearby. The structure and columns of our data files are explained in Tables 3 and 4. We zipped the files with a temporal resolution of 10 s and 1 min to decrease the file size. The size of each file varies between 110 MB and 9 GB, mostly depending on the temporal resolution. Metadata is available in a separate file called datapackage.json. A good example of how to read, restructure and plot the data with Python is available in the official code repository (please refer to the section Code Availability) in WPuQ/plots.py/WPuQPlots.plot_seasonal_load_curves. The free software HDFView is another great alternative to open and view HDF5 files.

## Data Availability

The code implementation was done in Python3. The scripts to perform the download, restructuring, validation and visualization of the data are available at the ISFH GitHub repository (https://github.com/ISFH/WPuQ).
